# Pyrolytic carbon coated black silicon

**DOI:** 10.1038/srep25922

**Published:** 2016-05-13

**Authors:** Ali Shah, Petri Stenberg, Lasse Karvonen, Rizwan Ali, Seppo Honkanen, Harri Lipsanen, N. Peyghambarian, Markku Kuittinen, Yuri Svirko, Tommi Kaplas

**Affiliations:** 1 Department of Micro and Nanosciences, Aalto University, Espoo, P.O. Box 13500, FI-00076, Finland; 2 Institute of Photonics, University of Eastern Finland, Joensuu, P.O. Box 111, FI-80101, Finland; 3 College of Optical Sciences, University of Arizona, 1630 E. University Blvd, Tucson, AZ 85721, USA

## Abstract

Carbon is the most well-known black material in the history of man. Throughout the centuries, carbon has been used as a black material for paintings, camouflage, and optics. Although, the techniques to make other black surfaces have evolved and become more sophisticated with time, carbon still remains one of the best black materials. Another well-known black surface is black silicon, reflecting less than 0.5% of incident light in visible spectral range but becomes a highly reflecting surface in wavelengths above 1000 nm. On the other hand, carbon absorbs at those and longer wavelengths. Thus, it is possible to combine black silicon with carbon to create an artificial material with very low reflectivity over a wide spectral range. Here we report our results on coating conformally black silicon substrate with amorphous pyrolytic carbon. We present a superior black surface with reflectance of light less than 0.5% in the spectral range of 350 nm to 2000 nm.

Creating a superior black surface has long been a lasting challenge for many decades^1^,^2^. Most promising results to-date in the visible (VIS) and ultra-violet (UV) spectral ranges have been achieved with black silicon (bSi), a silicon surface decorated with randomly distributed vertical pyramidal needles of micrometer size height and a very large aspect ratio^1^,^2^. Photons incident on the bSi surface experience multiple reflections on pyramids’ surfaces and photons with energies higher than the silicon bandgap are rapidly absorbed by silicon. Such a surface structure provides superior suppression of the reflectivity by three orders of magnitude in the VIS, making bSi a versatile platform for highly effective solar cells^3^,^4^ or THz emission sources^5^. However, the anti-reflectance performance of bSi reduces rapidly when the incident photon energy becomes lower than the silicon bandgap, i.e. the multiple reflections cannot provide the required energy loss^2^. In order to extend the absorbing range towards mid- and far-IR range the pyramidal needles that compose the bSi surface should be able to strongly absorb such photons while having the same aspect ratio and spatial distribution. In order to achieve that goal, a modification of bSi and deposition of broadband absorbing materials on bSi have been proposed^6^,^7^. Very recently it was observed that highly-doped bSi coated with a subwavelength thick oxide layer using atomic layer deposition technique is also capable to suppress reflectivity over a wide spectral range^8^,^9^. One may expect that an ultra-thin, strongly absorbing carbon layer which is conformally deposited on bSi can also be used to enhance its antireflection properties.

Among carbon based materials, *sp*^2^ hydrogenated carbon is well known for its absorption in a wide spectral range from UV to far IR due to the π-band electronic absorption. Quite recently, carbon nanotubes (CNTs) and graphene have demonstrated great capability to efficiently absorb electromagnetic radiation and have been suggested as perfect black materials^10^,^11^. However, CNTs and graphene pose serious challenges in fabrication since both of these materials typically require a catalyst substrate for the growth. Also, the deposition of these materials on nano- and microstructured surfaces like bSi is difficult task that makes their use in practical devices questionable.

Alternative route to improve bSi performance is to employ pyrolytic carbon (PyC), an amorphous carbon allotrope with dominating *sp*^2^ bonding. PyC - similar to carbon nanotubes (CNTs) and graphene - displays strong absorption of the EM radiation in a wide spectral range spanning from UV to far-IR^10^,^11^,^12^,^13^. It is conventionally produced by chemical vapor deposition (CVD) from gaseous hydrocarbon precursors at about 1000 °C^14^,^15^. The deposited PyC film consists of small, lamellar graphitic ribbons with size of several nanometers and relatively small amount of amorphous carbon^16^,^17^. Due to the dominating *sp*^2^ hydrogenation and π-electrons, it is expected to have strong optical absorption over a wide spectral band^18^. This property makes PyC films attractive for designing new types of black surfaces that can complement materials such as black silicon (bSi).

It has been recently demonstrated that CVD with light hydrocarbons (e.g. acetylene^16^ and methane^17^) can be employed not only for fabrication of carbon fiber-reinforced carbon but also for deposition of the nanometer thin PyC films on dielectric substrates. This experimental finding has opened a wide range of new applications of PyC including electrochemical measurements^16^, conductive vias through dielectric^19^, carbon-silicon Schottky-barrier diodes^20^ and shielding of the electromagnetic radiation in a wide spectral range^13^.

Despite the fact that amorphous carbon inclusions make electrical conductivity of PyC films somewhat lower than that of graphene^16^,^17^,^21^, the transmittance of a PyC film resembles that of graphene. In particular, the transmittance of PyC is near constant in the near-IR spectral range and a wide absorption peak is located at 270 nm, at the same wavelength as for graphene^12^,^22^. Moreover, catalyst free synthesis of PyC permits coating of metal and dielectric substrates of any shape.

In this paper, we demonstrate a novel superior black surface for the spectral range of UV to mid-IR that employs PyC-enhanced bSi structure. By developing an inexpensive and scalable technique we achieved conformally coated bSi surface with the PyC film thickness of 25 nm and demonstrated that such coating enormously suppresses reflectance in the IR. The obtained results allow us also to revisit the long-standing discussion on the mechanism of the CVD of PyC from light hydrocarbons. In particular, the demonstrated conformal growth on micro- and nanostructured substrates indicates that the surface activated growth process is the dominant mechanism of the PyC film synthesis.

For the demonstration of ultra-thin PyC formation on Si, we deposit 25 nm thick layer of PyC on a bare Si substrate. Figure 1a,b show the SEM images of the PyC layer grown on a bare Si substrate. The film grows uniformly all around the substrate covering the sample surface with a thin layer of PyC. Although, the PyC layer is only 25 nm thick, it has crucial impact on the reflectance of the Si. Specifically, one can observe from Fig. 1c that the deposition of ultra-thin PyC layer makes the reflectance nearly wavelength independent in the range from 400 nm to 2000 nm. In UV, however, the reflectance decreases rapidly due to the strong absorption in the PyC film. This absorption resembles the M-saddle point absorption of graphene and has its maximum around the same wavelength of 270 nm (see Supplementary information).

In the characterization of PyC with Raman measurements, the disorder induced D peak (1350 cm^−1^) and graphitic G mode (1600 cm^−1^) are conventionally used to characterize the crystallinity of the fabricated film^23^,^24^. The measured Raman spectrum shown in Fig. 1d (and Fig. S1, Supplementary information) has a weak *D’* mode at around 1500–1550 cm^−1^, which is a signature of the amorphous carbon^25^.

Moreover, in contrast to Raman spectrum of highly crystalline graphite and graphene, the 2D’ peak in PyC film, shown in Fig. 1d is greatly widened and significantly suppressed. Analysis of the Raman spectra (Supplementary information) allows us to conclude that in the deposited PyC film, the average in-plane lattice parameter is around 2.2 nm.

The exceptional conformality of the PyC coating facilitates deposition on nanostructured surfaces with sharp and randomly distributed surface features. In Fig 2a,c, one can observe a bSi without and with PyC, respectively. Similar to Si gratings (see Supplementary information), the PyC film covers the pyramidal silicon features with exceptional conformality offering negligible film surface roughness. In Fig. 2c one may observe that the PyC coating does not produce visible changes in bSi structure. This obtained high quality conformal PyC coating indicates that the surface of the PyC film remains active throughout the whole CVD process. Specifically, the PyC film keeps growing uniformly despite a few tens of nanometers gap between the nanostructures, see Fig. 2d (and Fig. S2 in the Supplementary information). This is a clear signature of dominating role of the reactive surface in the PyC deposition that leads to the highly uniform and conformal coating of the patterned substrate.

The reflectance of bSi after PyC coating, shown in Fig. 3a, changes drastically in the IR (λ > 1000 nm). Specifically, while bare bSi reflects more than 80% of the incident light from 1200 nm to 2000 nm (Fig. 3a), the reflection of bSi coated with PyC film is as low as 0.5% (Fig. 3c). With the wavelength higher than 2000 nm, the reflectance of thin PyC film coated bSi increases as the bSi microstructures are not deep enough for the corresponding wavelengths (see Supplementary information). It is worth noting that, although bare Si has lower reflectance compared to PyC coated Si in the VIS spectral range (Fig. 1), the anti-reflecting properties of PyC coated bSi in VIS-IR spectral range is superior to that of bare bSi (Fig. 3b).

The strong IR absorption of PyC coated bSi is very similar to the results obtained by using strongly doped silicon^9^, but the absorption mechanism is very different. The strong doping increases the number of free electrons in silicon and thus increases light absorption inside the silicon substrate. Since the bSi surface is acting as an anti-reflecting surface, the light is trapped and absorbed by the strongly doped silicon substrate. At light doping, the silicon itself does not absorb as seen from the reflectance spectrum in Fig. 3a. Instead, the absorbing carbon layer, which is only 25 nm thick, interacts with the incident light and absorbs it at the surface of the bSi substrate. Thus, the performance of the PyC-coated structure is actually not dependent on the absorption of the substrate.

Since the IR absorption in lightly doped silicon is relatively weak, the strong reflection at near IR originates from the backside reflection of the bare and black silicon substrate. In order to demonstrate this, we measured reflectance of black and bare Si with a spectralon disk as a backside reflector. This configuration highlights the influence of the 25 nm thick PyC film on the reflectivity. Specifically, we showed that both bare and black Si reflect about 80% and 50% of the incident IR radiation with and without backside reflector, respectively. However, the reflectance of the PyC coated samples measured with or without the spectralon disk was the same, which is an evidence of very strong IR radiation absorption in the ultra-thin PyC film. Although, in our experiment we did not measure the dependence of the reflectivity on the angle of incidence, we believe that the PyC coating should suppress reflection also at oblique incidence. This is because 25 nm thick PyC layer is thin enough not to affect to a few micron deep features on the bSi surface.

Since the in-plane size of intertwined graphene nanoribbons in PyC is about a few nanometers, strong electron scattering makes the electrical conductivity of PyC lower than that of graphene^16^,^17^,^21^ and also suppresses electro- and nonlinear-optical effects^12^. Thus one may expect that absorbance of graphene coated bSi will outperform PyC coated bSi. However, coating of the pyramidal bSi surface conformally by graphene is hardly achievable. Furthermore, monolayer thick graphene is not enough for sufficient absorbance. Meanwhile, PyC offers a reliable conformal coating method as 25 nm thick PyC layer doesn’t make changes to the structural appearance of bSi.

The conformality of PyC is attributed to the nature of the CVD process, where the active sites are randomly distributed over the surface. Hydrocarbon molecules on these active sites are absorbed followed by hydrogen desorption^26^. Thus, one may expect that this mechanism can result in synthesis of the solid PyC layer that will precisely follow the shape of a substrate. In the initial stage of the CVD process, the methane decomposes forming carbon dimers (C2) in gaseous atmosphere^14^,^26^,^27^. Interaction of dimers with other gaseous species in the CVD chamber leads to the formation of aromatic C6-based compounds by C2→C6 route (other routes are also possible^27^) that attach on the substrate forming new active sites for substrate-gas interaction. Specifically, when a massive aromatic species land on the substrate, they may continue evolving thus forming active surface sites that initiate growth of the carbon rings both at the edges and on the top of basal planes^26^. Such surface activated synthesis explains the conformal growth of PyC over the arbitrary shaped substrate surface.

In summary, we have demonstrated a wide band anti-reflecting surface by combining bSi with PyC thin film. The structural integrity of the substrate was not compromised and enhanced light absorption was demonstrated over a wide band. Absorption and Raman spectroscopic measurements confirm that the PyC film is uniform over the substrate and the structure is amorphous. One may expect a lower DC conductivity and optical nonlinearity compared with highly oriented graphite films and graphene^12^. However, the excellent uniformity and extreme conformality of PyC films over large areas indicates dominant heterogeneous growth mechanism. The advantages of PyC coating make it preferable and practical for many potential applications. In particular, biocompatibility and chemical inertness of PyC coated bSi suggest use in biomedical sensors and increase the sensitivity in e.g. electrochemical experiments, where the molecule adhesion plays a crucial role. Moreover, since an ultra-thin PyC film is robust and easily holds its weight^28^, it could be used as a membrane material for a free-standing gratings in e.g. micro- and nanoelectromechanical systems.

## Methods

### Sample preparation

In order to demonstrate the conformal PyC deposition, we coated a bare silicon substrate and bSi with PyC films. The bSi was formed in a cryogenic, inductively coupled plasma reactive ion etcher (ICP-RIE; Plasmalab System 100, Oxford Instruments, UK) using the following optimized parameters: 40 sccm SF_6_, 18 sccm O_2_, 6 W forward power, 1000 W ICP power, −110 °C, 10 mTorr pressure and helium backside cooling. Details of bSi fabrication are reported in previous studies^29^,^30^. The silicon used in the experiment was lightly p-doped, 0.5 mm thick silicon wafers.

The PyC coatings of the bare Si and bSi were performed by using a hot wall CVD system described in ref. 10. The system employs methane/hydrogen mixture as a carbon source and is equipped with a cylindrical quartz chamber, a tubular furnace (Carbolite CTF 12/75/700) and a computer controlled vacuum pump and gas flow controllers. The thickness of the fabricated PyC films is determined by the methane concentration in the hydrogen-methane gas mixture and pressure in the CVD chamber.

Before heating the oven, the CVD chamber was purged twice with nitrogen and once with hydrogen. In the first stage of the process, the chamber was heated up to 700 °C during one hour in hydrogen atmosphere (7 mbar). At 700 °C, it was pumped down to vacuum and the hydrogen was replaced with hydrogen-methane gas mixture. After the gas exchange the chamber was heated from 700 °C to 1100 °C in 40 min. At 1100 °C, the catalyst-free spontaneous methane decomposition starts. This process is initiated by methane radicals that form new hydrocarbon molecules, which are combined into massive polycyclic aromatic structures and deposited onto substrate^2^,^3^. After the process both sides of the substrate were coated with the PyC film. In our experiments we did not observe any dependence on the sample position inside the chamber, i.e. PyC films of the same thickness were deposited on the both sides of the horizontally and vertically oriented substrates. The gravity is not involved in the deposition process, i.e. it is unlikely dominated by landing of massive hydrocarbon particles on the substrates. The dependence of the deposited PyC film thickness on methane concentration can be found in ref. 17.

In order to demonstrate that developed technique can be employed for PyC deposition on arbitrary substrates, we also coated one-dimensional gratings fabricated from silica, silicon and TiO_2_ substrates (see Supplementary information).

### Characterization

The optical reflectance characterization for PyC was done by using spectrophotometer (Perkin Elmer lambda 1050 with 8/d geometry using spectral range from 350 nm to 2000 nm. Measurement was done using a spectralon disk as a backside mirror and about 1 mm × 10 mm beam spot size. Raman spectroscopy was done by Renishaw inVia Raman microscope with excitation wavelength of 514 nm. The excitation beam intensity was kept low in order to avoid heat induced phenomena in the carbon film. Scanning electron microscopy was done by SEM Zeiss Supra 40 and Leo 1550 Gemini.

## Additional Information

**How to cite this article**: Shah, A. *et al*. Pyrolytic carbon coated black silicon. *Sci. Rep*. **6**, 25922; doi: 10.1038/srep25922 (2016).

## Supplementary Material

Supplementary Information

## Figures and Tables

**Figure 1:**
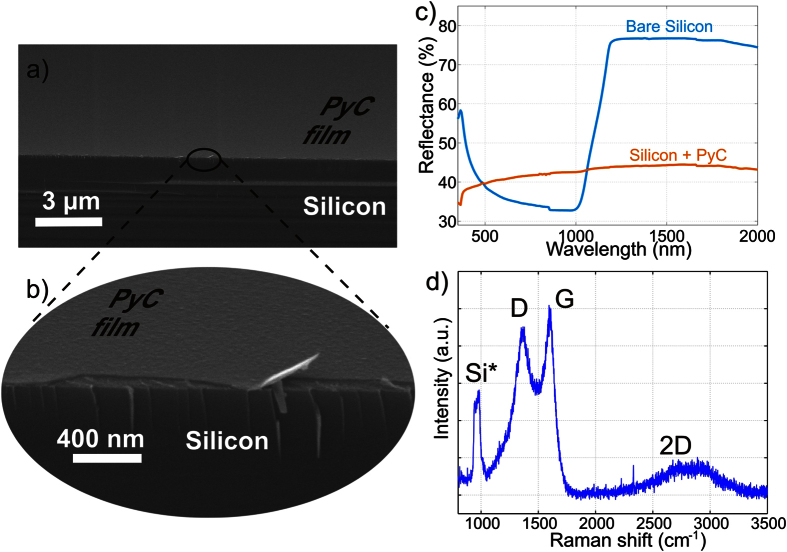
Scanning electron microscope (SEM) images and optical characterization of a PyC film grown on a bare Si substrate. (**a**) The film grows smoothly throughout the substrate surface. (**b**) High resolution SEM image shows the ultra-thin PyC film grown on surface of the Si substrate. (**c**) Reflectance of bare Si and Si coated with PyC in wavelength range from 350–2000 nm using a spectralon disk as a backside reflector. PyC coated Si reflects about 45% of the incident radiation through whole spectrum. It is interesting to note the drastic change in reflectance despite the carbon film thickness being only 25 nm (see also simulation in Supplementary information). (**d**) The measured Raman spectrum of the PyC film shows peaks at 1365 cm^−1^ and 1596 cm^−1^ typical for highly amorphous graphite carbon and very faint and wide 2D peak. (Si^*^ at about 1000 cm^−1^ is the second order silicon mode).

**Figure 2:**
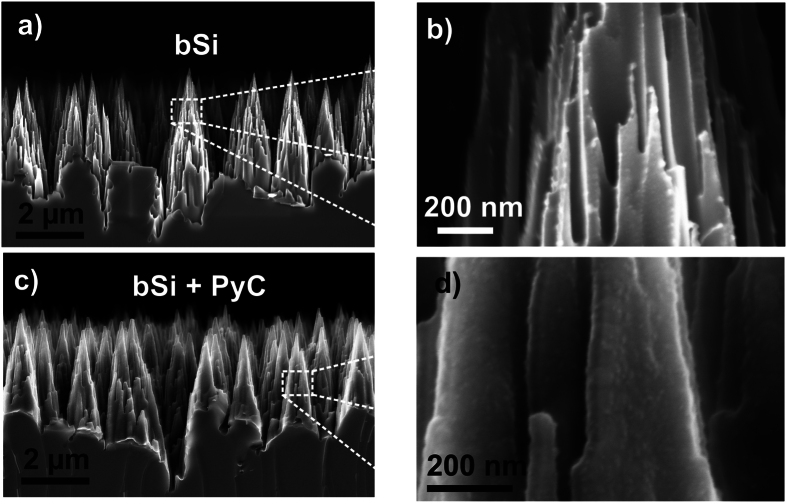
Black silicon surface with and without pyrolytic carbon. (**a**,**b**) Black silicon substrate consists of sharp silicon needles randomly distributed uniformly over the Si substrate. (**c**,**d**) After coating the sample with 25 nm thick PyC layer the topography of bSi is well preserved and even smallest details are evenly coated with PyC.

**Figure 3:**
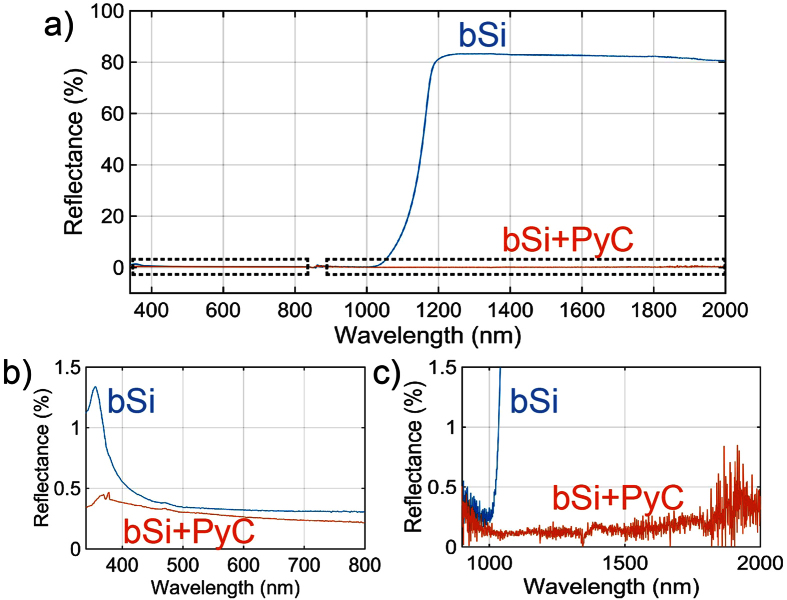
Reflectance of a bSi with and without PyC. (**a**) Reflectance spectra of bSi and PyC coated bSi. Similar to the bare Si, the bSi reflectance is about 80% at wavelengths above 1000 nm. However, coating of bSi with PyC reduces the reflectance down to less than 0.5%. (**b**) Although, bSi reflects less than 0.5% in the visible spectral range, there is a reflection peak around 375 nm where the reflectance becomes more than 1%. In PyC coated bSi this peak is suppressed while the absorption stays higher than 99.5%. (**c**) bSi coated with PyC has reflectance less than 0.5% in the 1200 nm to 2000 nm spectral range while the reflectance of uncoated bSi is more than 80%.
