# Supplementary material for: Pyrolytic carbon coated black silicon
Source: Sci Rep. 2016 May 13;6:25922. doi: 10.1038/srep25922 (PMC4865946; doi:10.1038/srep25922)
Supplement: Supplementary Information [file srep25922-s1.doc]

**Supplementary information**

Pyrolytic carbon coated black silicon

Ali Shah,1 Petri Stenberg,2 Lasse Karvonen,1 Rizwan Ali,2 Seppo Honkanen,2 Harri Lipsanen,1 N. Peyghambarian,1,2,3 Markku Kuittinen,2 Yuri Svirko,2 and Tommi Kaplas 2,*

*1Department of Micro and Nanosciences, Aalto University, Espoo, P.O. Box 13500, FI-00076, Finland*

*2Institute of Photonics, University of Eastern Finland, Joensuu, P.O. Box 111, FI-80101, Finland*

*3College of Optical Sciences, University of Arizona, 1630 E. University Blvd, Tucson, AZ 85721, USA*

**­tommi.kaplas@uef.fi*

In order to demonstrate the conformality of PyC deposition process, we coated with PyC films a bare SiO2 substrate and dielectric gratings of various shapes and periodicity. The gratings with sub-micron periodicity were fabricated on fused silica, silicon and TiO2 by using e-beam lithography technique. The TiO2 substrate was 250 nm thick layer on a planar silica substrate obtained by physical vapor deposition. The SiO2 and TiO2 substrates were coated with thin layer of chromium to prevent charging during e-beam exposure. The substrates were spin coated with high resolution resists (HSQ for the Si substrate and ZEP 7000-22 for SiO2 and TiO2/SiO2) and patterned by e-beam writer (Vistec EBPG-5000). After the e-beam exposure and subsequent development, the samples were etched by reactive ion etching and the resist and chromium masks were removed. The period and height of grating structures were 200 nm and 250 nm, respectively. The SEM images of the fabricated gratings are shown in Fig. S2.

The PyC coatings of the fabricated gratings were performed by using a conventional hot wall CVD system. The system employs methane/hydrogen mixture as a carbon source and is equipped with a cylindrical quartz chamber, a tubular furnace (Carbolite CTF 12/75/700) and a computer controlled vacuum pump and gas flow controllers. The thickness of the fabricated PyC films is determined by the methane concentration in the hydrogen-methane gas mixture and pressure in the CVD chamber.

Before heating the oven, the CVD chamber was purged twice with nitrogen and once with hydrogen. In first stage of the process, the chamber was heated up to 700 °C during one hour in hydrogen atmosphere (7 mbar). At 700 °C, it was pumped down to vacuum and the hydrogen was replaced with hydrogen-methane gas mixture. After the gas exchange the chamber was heated from 700 °C to 1100 °C in 40 min. At 1100 °C, the catalyst-free spontaneous methane decomposition starts. After the process both sides of the substrate were coated with the PyC film. For optical measurements backside PyC was removed by reactive ion etching using oxygen plasma (2 min / 20 sccm / 100 W).

The optical characterization of the PyC film grown on bare SiO2 substrate (shown in Fig. S1a and Fig. S1b) was done by transmission spectroscopy and Raman spectroscopy. The transmission of the PyC film was measured with Perkin-Elmer lambda-18 spectrophotometer using normal incidence. Before the transmission measurement the setup was calibrated with plain quartz substrate. The Raman measurement was done by inVia Raman Microscope, with 514 nm excitation wavelength. The excitation beam intensity was kept low in order to avoid heat induced phenomena in the carbon film.


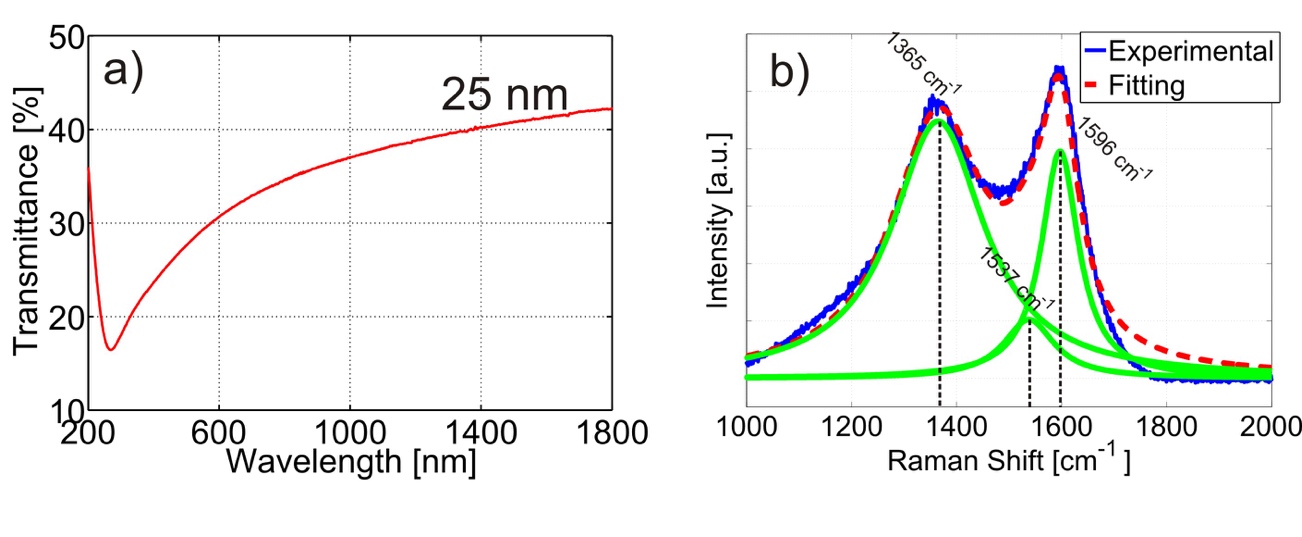


Figure S1. (a) The transmittance spectrum of the 25 nm thick PyC film deposited on the SiO2 substrate. One can observe that the spectrum resembles that of a multilayered graphene except of a broader the absorption peak at 270 nm. (b) The measured (blue line) Raman spectrum of the PyC film dominates peaks at 1365 cm-1 and 1596 cm-1 typical for highly amorphous graphite carbon. This spectrum can be well reproduced by fitting (red line) with three Lorentzian resonances centered at 1365, 1537 and 1598 cm-1 which are shown by green lines.

Figure S1a shows the transmittance of the 25 nm thick film in spectral range from 200 nm to 1800 nm. One can observe that the transmittance spectrum resembles that of a few layered graphene yet having broader absorption peak near 270 nm. Peak broadening can be explained by the amorphous nature of the PyC if one recall that the absorption resonance centered at about 270 nm is attributed to the M-saddle point in the graphite band structure [1]. Since PyC film consists of nanosized graphene flakes, the band structure of each flake in the vicinity of the M-saddle point depends on the flake size, shape and orientation. This makes the shape of the resonance different from that in crystalline graphite. Moreover, the pronounced electron scattering on the flake boundaries and grains of the amorphous carbon broadens the absorption resonance at 270 nm even further.

In the characterization of carbon materials with Raman spectra measurements, the disorder induced *D* mode and graphitic *G* mode are conventionally used to characterize the crystallinity of the fabricated film. The blue line in Fig. S1b shows the Raman spectra of the PyC film. This spectrum can be reproduced by fitting (red line in Fig. S1c) with three Lorentzian peaks centered at 1365 cm-1, 1537 cm-1 and 1598 cm-1 (green lines) that represent *D*, *D’’* and *G* modes, respectively [2,3]. It is worth noting that in the Raman spectrum, a weak *D”* mode at around 1500 – 1550 cm-1 is a signature of the amorphous carbon [3].

The position and FWHM of the *G* peak (about 1600 cm-1 and 65 cm-1, respectively) as well as a weak resonance at 1537 cm-1 indicate that the PyC film is highly amorphous. In order to estimate the in-plane correlation length (*La*) of graphitic crystals in PyC one can employ the Ferrari-Robertson (FR) relation [4]:

$L_{a}^{2}(nm)=\frac{1}{C(\lambda)}\frac{I(D)}{I(G)}$, (1)

where, *I(D)* and *I(G)* are intensities of the *D* and *G* peaks in the Raman spectra, respectively, while *C(λ)* depends on the excitation wavelength *λ*. In particular, *C*(*λ =* 514 nm) = 0.55 [4]. By substituting that into Eq. (1) one can conclude, from the results of the Raman measurement, that in the fabricated PyC film, *La* ≈ 2.2 nm. Since it is the average size of the arbitrary shaped graphene flakes in the film, one may expect that the size of nanocrystals does not exceed 5 nm.

In order to demonstrate conformal carbon coating, we performed deposition of the PyC films on SiO2, Si and TiO2 gratings. By choosing different substrate materials we aim to demonstrate that the substrate material does not significantly affect to the conformal growth of PyC. The Si and TiO2 gratings were coated with the 25 nm thick PyC film, while the SiO2 grating was coated with the film with thickness of 50 nm in order to visualize the effect of the film thickness on the conformal deposition. After the CVD, the samples were cleaved and characterized with SEM.


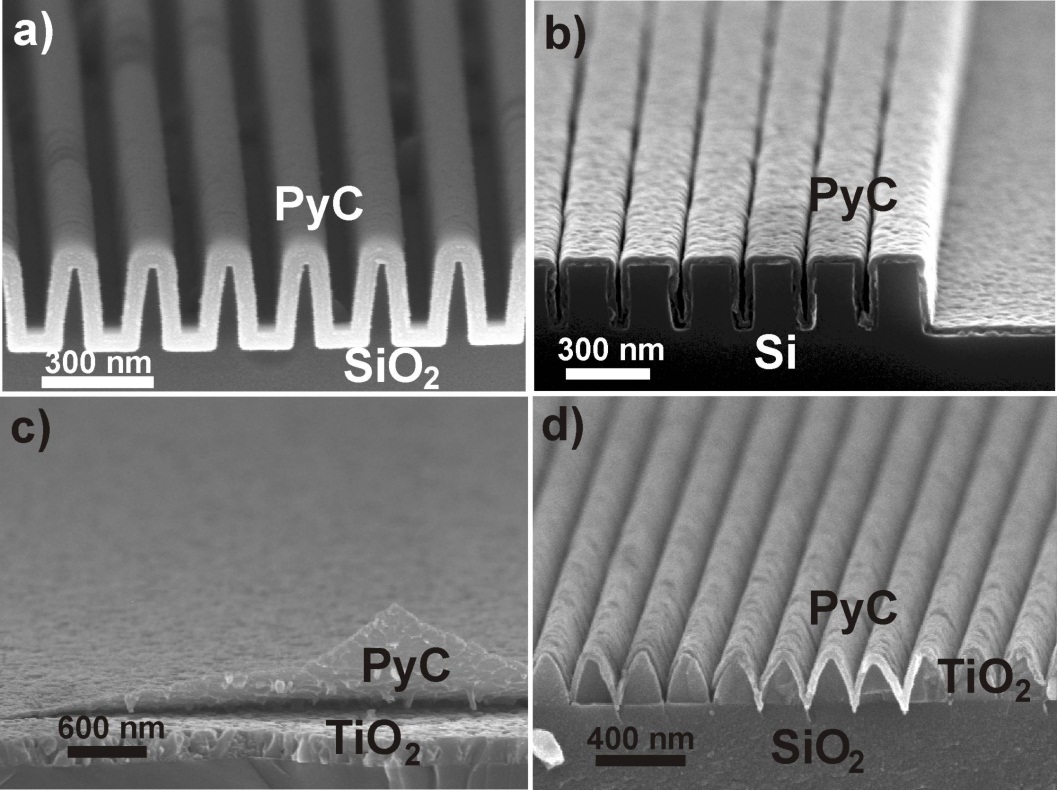


Figure S2. (a) A SiO2 grating with period of 200 nm coated with 50 nm thick PyC film. (b) 25 nm thick PyC film grown on a silicon grating with period of 200 nm. One may observe that despite the gap between grating lines is suppressed to only a few tens of nanometers, there are no signs that carbon layer would deviate from conformal growth style. (c) and (d) 25 nm thick PyC deposited on a bare TiO2 coatedSiO2 substrate and TiO2 grating structure.

Furthermore, we simulated reflection of a bare silicon substrate with and without PyC film by using well-known equation [5]:

$R=\left|\frac{r_{12}+r_{23}\exp(2i\beta)}{1+r_{12}r_{23}\exp(2i\beta)}\right|^{2}$, (2)

where at normal incidence, *r12=(1-nPyC)/(1+nPyC)* and *r23=(nPyC-nSi)/(nPyC+nSi)* are amplitude reflection coefficients of the air-PyC and PyC-Si interfaces, *nSi* and *nPyC* are refractive indices of silicon and pyrocarbon [6], respectively, *β = 2πnPyCh/λ, h* is the thickness of PyC and *λ* is the wavelength of the light. These simulated results are presented in Fig. S3.


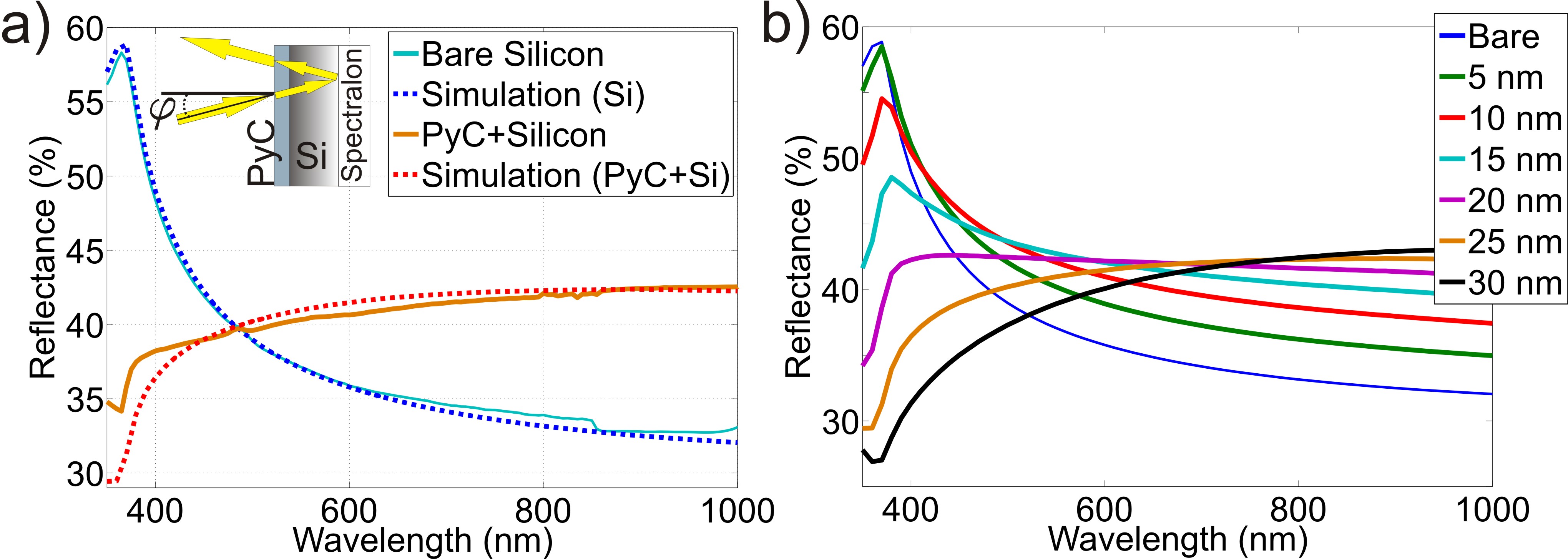


Figure S3. Measured and simulated reflectance of a bare silicon with and without PyC film. (a) At the spectral range from 350 nm to 1000 nm the reflectance is dominated by the front side interface reflectance while at above 1000 nm the backside reflection plays an important role when Si becomes highly transparent (in our experiment we used a spectralon disk as a backside reflector - inset). Even if the PyC film thickness is only 25 nm, it has a crucial impact to the reflectance of a silicon substrate. (b) Simulation shows that only a 10 nm – 15 nm thick PyC film has a huge impact to the reflectance from a bare silicon substrate.

**References**

1 Roberts, A.T.et al. Phys. Rev. Lett. **112**, 187401 (2014).

2 Reich, S. & Thomsen, C. Raman spectroscopy of graphite, Phil. Trans. R. Soc. Lond. A 362, 2271−2288 (2004).

3 Jawhari, T., Roid, A. & Casado, J. Raman spectroscopic characterization of some commercially available carbon black materials. Carbon 33(11), 1561−5 (1995).

4 Ferrari, A.C. & Robertson, J. Interpretation of Raman spectra of disordered and amorphous carbon Phys. Rev. B 61(20), 14095−13 (2000).

5 Born, M. & Wolf, E., *Principles of Optics, 4th edition, Ch. 1.6, 61-66* (Pergamon Press, 1999).

6 Kaplas, T. et al. Nonlinear refraction in semitransparent pyrolytic carbon films. Opt. Mater. Express 2, 1822−1827 (2012).
